# Fragmented QRS as a risk marker for the occurrence of ventricular fibrillation in patients with variant angina

**DOI:** 10.1111/anec.12937

**Published:** 2022-02-16

**Authors:** Tetsuji Shinohara, Keisuke Yonezu, Kei Hirota, Hidekazu Kondo, Akira Fukui, Hidefumi Akioka, Yasushi Teshima, Kunio Yufu, Mikiko Nakagawa, Naohiko Takahashi

**Affiliations:** ^1^ 12995 Department of Cardiology and Clinical Examination Faculty of Medicine Oita University Oita Japan

**Keywords:** fragmented QRS, risk stratification, variant angina, vasospastic angina, ventricular fibrillation

## Abstract

**Background:**

Variant angina (VA) is caused by reversible coronary artery spasm, which is characterized by chest pain with ST‐segment elevations on standard 12‐lead electrocardiogram (ECG) at rest. Ventricular fibrillation (VF) is often caused by VA attack, but the risk stratification is not well understood. The purpose of this study was to evaluate the impact of fragmented QRS (fQRS) on VF occurrence in VA patients.

**Methods:**

Ninety‐four patients who showed ST elevation on 12‐lead ECGs with total or nearly total occlusion in response to coronary spasm provocation test were enrolled. Among them, 16 patients had documented VF before hospital admission (*n* = 12) or experienced VF during provocation test (*n* = 4) (VF occurrence group). The fQRS was defined as the presence of spikes within the QRS complex of two or more consecutive leads.

**Results:**

The prevalence of fQRS was more often observed in the VF occurrence group than in the non‐VF occurrence group (63% [10/16] vs. 27% [21/78], *p* = 0.009). Univariate analyses revealed that age, history of syncope, QTc, and the presence of fQRS were associated with VF occurrence (*p* = 0.004, 0.005, 0.029, and 0.008, respectively). Furthermore, upon multivariate analyses using those risk factors, age, QTc, and fQRS predicted VF occurrence independently (*p* = 0.007, 0.041, and 0.014, respectively).

**Conclusions:**

The present study demonstrated that fQRS in VA patients is a risk factor for VF. The fQRS may be a useful factor for the risk stratification of VF occurrence in VA patients.

## 1 INTRODUCTION

Variant angina (VA) is caused by reversible coronary artery spasms. It is characterized by chest pain with ST‐segment elevation on a 12‐lead electrocardiogram (ECG) at rest and is not triggered by daytime exercise (JCS Joint Working Group, 2014). Hence, VA is generally considered to be severe vasospastic angina (VSA). The prognosis for VSA is relatively benign as long as patients remain on calcium channel blockers and avoid smoking (Bory et al., 1996; JCS Joint Working Group, 2014). However, coronary artery spasm may also have an important role in the pathogenesis of ventricular arrhythmia and subsequent cardiac arrest (Myerburg et al., 1992). Although an implantable cardioverter–defibrillator (ICD) is required to prevent sudden cardiac death in high‐risk patients of lethal ventricular arrhythmia, the indication for ICD implantation for a patient with coronary spasm‐induced ventricular fibrillation (VF) remains debatable (Matsue et al., 2012; Yamashina et al., 2014).

Fragmented QRS (fQRS) is defined as additional spikes within the QRS wave, and it has been known to be a marker of myocardial dysfunction resulting in an intraventricular condition defect. Therefore, it is believed that the fQRS wave can be caused by a zig‐zag condition around the dysfunctional myocardium, (Das et al., 2010) resulting in multiple spikes within the QRS complex. Additionally, the myocardial scar detected by the fQRS wave is strongly associated with ventricular dysfunction and is a substrate for reentrant ventricular arrhythmia including VF. (Pietrasik & Zareba, 2012) Furthermore, the fQRS has been postulated to be predictive of outcomes in various organic heart diseases, such as ischemic heart disease, (Luo et al., 2020) dilated cardiomyopathy, (Marume et al., 2021) arrhythmogenic right ventricular cardiomyopathy, (Peters et al., 2008) sarcoidosis, (Schuller et al., 2011) Brugada syndrome (Morita et al., 2017) and acquired long QT syndrome. (Haraoka et al., 2010) Nevertheless, whether the fQRS is involved in the occurrence of VF in VA patients who are not considered to have organic cardiac dysfunction remains unclear. In the present study, we investigated the association between the fQRS on 12‐lead ECG and coronary spasm‐induced VF occurrence.

## 2 METHODS

### 2.1 Study design and patients

The study protocol was approved by the ethics committee of the Oita University Hospital (Yufu, Japan). All patients provided their written informed consent to participate in this study.

This retrospective observational study was conducted between January 2007 and August 2021. It involved 698 consecutive patients who underwent coronary angiography examinations for the diagnosis of VSA in our hospital. The patients demonstrated chest pain at rest, but 12‐lead ECGs were not recorded during this episode. They underwent coronary angiography with a coronary spasm provocation to facilitate a diagnosis. The protocol of the coronary spasm provocation test (CSPT) was as follows: All vasodilator drugs and calcium antagonists were discontinued ≥3 days before coronary angiography. After confirming no significant stenotic lesions in the right and left coronary arteries, the patients underwent a CSPT. We injected 30 µg of ergonovine solution in physiologic saline into the right coronary artery for 2 min and performed coronary angiography 2 min after the completion of the injection. In case of a negative result in the provocation test, we proceeded to the left coronary provocation with 50 µg of ergonovine test after 5 min. VA was defined as patients who showed transient ST‐segment elevation on 12‐lead ECGs during total or nearly total (99% stenosis with delay) occlusion by an ergonovine‐induced CSPT. If VF was induced in the right coronary artery, the CSPT was not performed in the left coronary artery. In patients with a history of VF, the CSPT was terminated as soon as coronary spasm was confirmed. Ninety‐four patients were diagnosed with VSA via a provocation test for coronary artery spasms according to the Guidelines for the Diagnosis and Treatment of Patients with Vasospastic Angina set by the Japanese Circulation Society (VA patients; JCS Joint Working Group, 2010). No patient had organic heart disease based on physical examination, chest radiography, 12‐lead ECG, echocardiography, treadmill exercise ECG, and ^201^Tl cardiac scintigraphy. We defined VSA and VA as follows: (i) VSA was diagnosed by transient ST elevation or depression of more than 0.1 mV or a new appearance of negative U‐wave on 12‐lead ECG during angina‐like attacks (JCS Joint Working Group, 2010). (ii) VA was diagnosed by rest angina, transient ST elevation, and improvement/resolution with sublingual nitrates based upon Prinzmetal's clinical criteria for diagnosing VA (Prinzmetal et al., 1959).

### 2.2 ECG analyses

**FIGURE 1:**
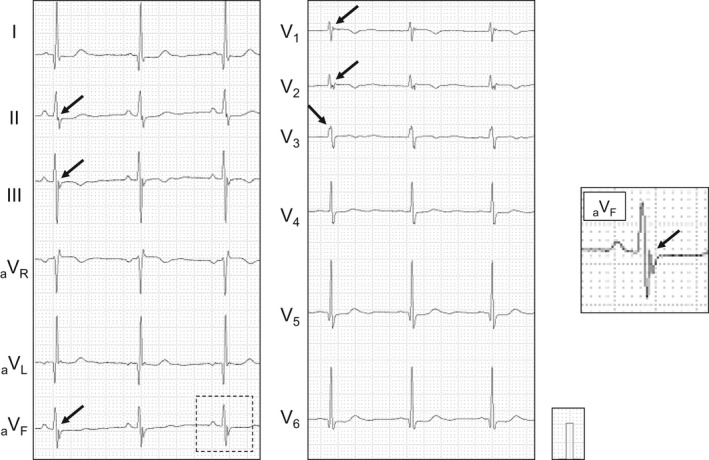
Representative electrocardiogram (ECG) of fragmented QRS (fQRS). The ECG shows fQRS distributing in anterior (V1‐3) and inferior (II, III, and aVF) segments. Arrows indicate spikes within QRS complex

We recorded standard 12‐lead surface digital ECGs using Cardiofax (Nihon Kohden) with a 150 Hz low‐pass filter with the subject lying supine. The fQRS wave on 12‐lead ECG was defined as the presence of spikes within the QRS complex (QRS <120 ms) according to the ECG criteria for fQRS (Figure 1; Das et al., 2010). Presence of fQRS was defined as fQRS in two contiguous leads corresponding to major myocardial segments: anterior (V1–V5), inferior (II, III, and aVF), or lateral (I, aVL, and V6; Das et al., 2010), To evaluate the association between fQRS severity and VF occurrence, we quantified fQRS extent based on the number of myocardial segments with fQRS. Single fQRS was defined as fQRS present in either the anterior, inferior, or lateral segment. Multiple fQRS was defined as the presence of fQRS in ≥2 myocardial segments. (Morita et al., 2017) The corrected QT interval (QTc) was evaluated using the Bazett formula. The standard and high intercostal (second and third intercostal) ECGs were recorded to exclude Brugada syndrome. This study did not include any patients with type 1 Brugada ECG (coved‐type ST elevation) in the right precordial leads.

All ECG recordings were analyzed at 400% size, and each parameter and the fragmentation of the QRS complex was measured by two experienced cardiologists blinded to ECG information (TS and KY).

### 2.3 Statistical analyses

Data are presented as the mean ±SD. Fisher's exact test was used for categorical variables. Statistical analyses were performed using the unpaired *t* test. The Wilcoxon signed‐ranks test was used for nonparametric data; *p* < 0.05 was considered significant. Univariate and multivariate logistic regression analyses were conducted to identify independent predictors of VF occurrence among the factors related to cardiac‐related sudden death. Multivariate logistic regression analyses were used only for variables with a significant univariate impact. All computations were conducted using JMP v9.0.0 (SAS) running under Windows™ 10 (Microsoft).

## 3 RESULTS

### 3.1 Patient characteristics

Table 1 shows the clinical characteristics of the VF occurrence group (*n* = 16) and the non‐VF occurrence group (*n* = 78). The VF occurrence group consisted of 12 VA patients with documented VF before hospital admission and four patients whose VF was induced for the first time during provocation testing. Patients with VF occurrence were significantly younger than non‐VF occurrence cases (*p* = 0.002). Additionally, the prevalence of history of syncope in the VF occurrence group was significantly higher than that of the non‐VF occurrence group (*p* = 0.007). By contrast, the body mass index in the VF occurrence group was not significantly different when compared with that of the non‐VF occurrence group; however, it tended to be lower (*p* = 0.07). Finally, there was no significant difference between the two groups regarding any other factor.

**TABLE 1:** Clinical characteristics of patients with and without VF

	VF (+) (*n* = 16)	VF (−) (*n* = 78)	*p* value
Male (%)	13 (81)	66 (85)	0.72
Age (years)	60 ± 8	69 ± 10	0.002**
BMI (kg/m^2^)	23.1 ± 3.4	25.0 ± 3.7	0.07
Family history of SCD	0 (0)	1 (1)	1.00
History of syncope	5 (31)	4 (5)	0.007**
Smoking	13 (81)	48 (62)	0.16
Brinkman index	462 ± 375	559 ± 437	0.45
Organic stenosis (≥90% stenosis)	1 (6)	12 (15)	0.45
Hypertension	8 (50)	35 (45)	0.79
Diabetes	1 (6)	16 (21)	0.29
LVEF (%)	60 ± 11	64 ± 10	0.13
LVDd (mm)	49 ± 9	47 ± 7	0.55

Abbreviations: BMI, body mass index; LVDd, left ventricular end‐diastolic diameter; LVEF, Left ventricular ejection fraction; SCD, sudden cardiac death; VF, ventricular fibrillation.

**p<0.01. VF occurrence group (*n* = 12) includes patients with a history of VF (*n* = 8).

### 3.2 Electrophysiologic characteristics

**FIGURE 2:**
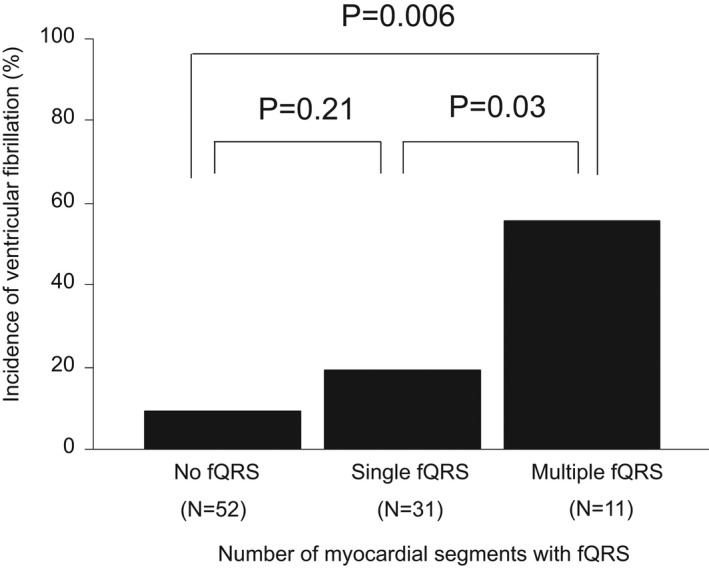
Relationship between the number of myocardial segments with fragmented QRS and the incidence of ventricular fibrillation. As the number of myocardial segments with fQRS increases, the incidence of ventricular fibrillation significantly increases as well (No fQRS vs. Multiple fQRS, *p* = 0.006; Single fQRS vs. Multiple QRS, *p* = 0.03). fQRS, fragmented QRS

Table 2 shows the 12‐lead ECG findings in the VF and non‐VF occurrence groups. The prevalence of fQRS was more often observed in the VF occurrence group than in the non‐VF occurrence group (*p* = 0.009). More specifically, the presence of fQRS in the VF occurrence group was observed in three cases (19%) in the anterior myocardial segment, in nine cases (56%) in the inferior myocardial segment, and in four cases (25%) in the lateral myocardial segment, respectively. The number of myocardial segments with fQRS in the VF occurrence group was not observed in any of the five cases, but it was observed in the single segment in six cases and in multiple segments in five cases, respectively. Furthermore, it was significantly more than in the VF occurrence group when compared with the non‐VF occurrence group (*p* = 0.01). Finally, as the number of fQRS segments increased, the incidence of VF also significantly increased (Figure 2).

**TABLE 2:** ECG findings of patients with and without VF

	VF (+) (*n* = 16)	VF (–) (*n* = 78)	*p* value
HR (bpm)	72 ± 16	65 ± 12	0.14
QRS (ms)	100 ± 15	101 ± 17	0.92
QTc width (ms)	436 ± 33	419 ± 28	0.07
Fragmented QRS, (%)	10 (63)	21 (27)	0.009**
Myocardial segments with fQRS			
Anterior myocardial segment	3 (19)	11 (14)	0.70
Inferior myocardial segment	9 (56)	21 (27)	0.04*
Lateral myocardial segment	4 (25)	7 (9)	0.09
Number of myocardial segments with fQRS	1.00 ± 0.82	0.50 ± 0.72	0.01*

Abbreviations: Fqrs, fragmented QRS; HR, heart rate; VF, ventricular fibrillation.

*p<0.05, **p<0.01. VF occurrence group (*n* = 16) includes patients with documented VF before hospital admission (*n* = 12).

### 3.3 Univariate and multivariate predictors of VF occurrence

Table 3 presents the results of univariate and multivariate logistic regression analyses of VF occurrence in VA patients. Univariate analyses revealed that age, history of syncope, QTc, and the presence of fQRS were associated with VF occurrence. Upon multivariate analyses, age (odds ratio [OR] = 0.910; 95% confidence interval [CI]: 0.849–0.975; *p* = 0.007), QTc (OR =1.026; 95% CI: 1.001–1.052; *p* = 0.041), and fQRS (OR =5.877; 95% CI: 1.431–24.14; *p* = 0.014) as risk factors could independently predict VF occurrence.

**TABLE 3:** Univariate and multivariate predictors of VF occurrence

	Univariate *p* value	Multivariate OR	95%CI	*p* value
Gender	0.7421			
Age	0.0038**	0.910	0.849 – 0.975	0.007**
BMI	0.0604			
History of syncope	0.0049**	5.522	0.909 – 33.54	0.063
Smoking	0.1167			
Organic stenosis	0.2925			
QTc	0.0287*	1.026	1.001 – 1.052	0.041*
Fragmented QRS	0.0075**	5.877	1.431 – 24.14	0.014*

BMI, body mass index; QTc, corrected QT interval; VF, ventricular fibrillation.

*p<0.05, **p<0.01.

### 3.4 Follow‐up

All patients with VA were treated with a calcium channel blocker and/or nitrovasodilator. Moreover, eight patients received ICD implantation to prevent sudden death. The clinical course was a follow‐up period of 67 ± 49 months. One patient without ICD implantation experienced sudden cardiac death 1 month after CSPT, and one VF episode was detected in a patient with ICD implantation.

## 4 DISCUSSION

### 4.1 Major findings

The present study elicited three main findings. First, VA patients with VF occurrence had more fQRS findings on 12‐lead ECGs than those with non‐VF occurrence. Second, the more widely the fQRS findings were in the myocardial segments, the higher the incidence of VF in VA patients. Third, multiple regression analysis showed that fQRS in VA patients could be a predictor of VF occurrence. To our knowledge, the present study is the first to demonstrate that the fQRS finding in VA patients is a risk factor of VF occurrence.

### 4.2 Variant angina

Since Prinzmetal et al. first reported on coronary spasm‐induced VA in 1959, several studies have revealed the clinical characteristics and outcomes of this disorder (Prinzmetal et al., 1959). Several prognostic factors for a coronary spasm, such as smoking, organic coronary stenosis, multivessel spasm, and the history of out‐of‐hospital cardiac arrest, have been established. Matsue et al. (2012) reported that lethal ventricular arrhythmia in patients with VSA tends to cause the recurrence of cardiopulmonary arrest. Hence, the authors recommended ICD implantation with medication in patients with VSA and lethal ventricular arrhythmia. Conversely, Yamashina et al. (2014) reported that appropriate medical treatment could achieve favorable long‐term outcomes even for patients with VSA associated with cardiac arrest. Deciding whether to implant an ICD in the event of encountering a patient with coronary spasm‐induced VF or not is usually not easy. Thus, the risk of VF occurrence in VA patients must be appropriately evaluated, and we should find out new predictors of VF occurrence in VA patients. The present study revealed that the existence of fQRS in VA patients could be a predictor of VF occurrence. Here, we propose that fQRS findings on 12‐lead ECGs may be one of the useful factors for the indication of ICD implantation in patients with coronary spasm‐induced VF.

### 4.3 Fragmented QRS

Our results indicated that the existence of fQRS plays an important role in the occurrence of VF in VA patients. Previous studies have shown that the existence of fQRS is a prognostic factor for patients with various organic heart diseases (Haraoka et al., 2010; Luo et al., 2020; Marume et al., 2021; Morita et al., 2017; Peters et al., 2008; Schuller et al., 2011). Das et al. found a correlation between fQRS and the presence of a myocardial scar. (Das et al., 2006) The authors reported that fQRS had a higher diagnostic sensitivity and negative predictive value for old myocardial infarction than a pathologic Q wave. This suggests that the pathogenesis of fQRS is associated with myocardial scarring due to a prior ischemic injury, which results in abnormal ventricular activation and impaired electrical activity on the ECG. Thus, fQRS is thought to represent heterogeneous activation of the ventricles due to the existence of myocardial scar and/or ischemia. The presence of myocardial scars and/or ischemia probably causes localized conduction blocks, leading to additional R′, notching of the S wave, or notching of the R wave. Hence, findings of fQRS, which represent abnormal electrical conduction, suggest that fatal reentrant ventricular arrhythmias and sudden cardiac death may be more likely to occur. By contrast, Morita et al. (2008) reported that fQRS is a marker for the substrate for spontaneous VF in patients with Brugada syndrome. Additionally, regarding the generation mechanism of fQRS, they demonstrated that activation delay in the epicardium could reproduce similar fQRS in the transmural ECG using canine right ventricular tissues. Accordingly, they proposed that fQRS might represent delayed conduction on the epicardium in patients with Brugada syndrome. The same group thereafter demonstrated that the progression of fQRS appearance was associated with the occurrence of VF in patients with BrS (Morita et al., 2017). Additionally, our laboratory recently reported that fQRS is also involved in VF occurrence in patients with early repolarization syndrome, which is similar to Brugada syndrome (Yonezu et al., 2021). The findings in the present study are consistent with previous reports that fQRS is one of the risk factors for the occurrence of lethal ventricular arrhythmias.

### 4.4 Risk factors of VF other than fragmented QRS

Besides fQRS findings, this study revealed that age and long QT interval are independent predictors of VF occurrence. Fumimoto et al. (Fumimoto et al., 2017) have also reported that VF survivors with VSA were significantly younger than non‐VF patients with VSA. We cannot explain the detailed mechanism by which young VA patients are susceptible to VF. Generally, the age distribution of patients suffering from coronary spasms is relatively lower than those with stable‐effort angina. (Kawano & Ogawa, 2004) This suggests that young age may tend to cause VF because of stronger or multiple coronary spasms. Furthermore, Kawana et al. (Kawana et al., 2013) reported that cardiac events occurred significantly more often in young women than in older women and lower age is a negative prognostic factor in female VSA patients.

Risk stratifications of VF have been clinically evaluated in patients with VSA. Nishizaki et al. (Nishizaki, 2017) reviewed that the inducibility of polymorphic ventricular tachycardia or VF in electrophysiologic study, increased QT dispersion, T‐wave alternans, and early repolarization during the asymptomatic period are considered factors for lethal ventricular arrhythmias during a coronary spasm. Additionally, it suggested that repolarization abnormality is involved in the risk of VF occurrence in VSA patients. Our study also demonstrated that prolonged QT interval, which represents repolarization abnormality, is one of the independent predictors of VF occurrence. This suggests that repolarization abnormality determined via QT prolongation, as well as depolarization abnormality indicated by fQRS, is one of the risk factors for VF occurrence in VA patients.

### 4.5 Study limitations

Our study has three main limitations. First, we evaluated a relatively small number of patients, especially cases with VF. The small number of patients might limit the interpretation of the results, although the results of the present study are not significantly different when compared with the results from previous studies. Second, we could not fully exclude the possibility of selection bias because the present study was retrospective and from a single center. The clinical implications of fQRS findings on 12‐lead ECGs in VA patients must be investigated further in large‐scale prospective studies. Third, multiple coronary spasms are highly dangerous and might induce VF. In the present study, however, we could not evaluate the association between VF occurrence and the incidence of multiple coronary spasms because further CSPT was aborted when VF was induced.

### 4.6 Conclusions

The present study demonstrated that fQRS on 12‐lead ECGs in VA patients is a risk factor for VF occurrence. The fQRS may be a useful factor for risk stratification of VF occurrence in VA patients.

## CONFLICT OF INTEREST

None.

## AUTHOR CONTRIBUTIONS

Dr. Shinohara acquired, analyzed, interpreted the data, and drafted the manuscript. Drs. Yonezu, Hirota, Kondo, Fukui, Akioka, Teshima, Yufu and Nakagawa assisted in acquiring, analyzing the data and interpreting results. Dr. Takahashi conceived and designed the research as the supervision. All co‐authors have read and approved the submission of this manuscript.

## ETHICS STATEMENT

The study protocol was approved by the ethics committee of the Oita University Hospital (Yufu, Japan). All patients provided their written informed consent to participate in this study.

## Data Availability

The data that support the findings of this study are available from the corresponding author upon reasonable request.

## References

[anec12937-bib-0001] Bory, M. , Pierron, F. , Panagides, D. , Bonnet, J. L. , Yvorra, S. , & Desfossez, L. (1996). Coronary artery spasm in patients with normal or near normal coronary arteries. Long‐term follow‐up of 277 patients. European Heart Journal, 17, 1015–1021. 10.1093/oxfordjournals.eurheartj.a014996 8809518

[anec12937-bib-0002] Das, M. K. , Khan, B. , Jacob, S. , Kumar, A. , & Mahenthiran, J. (2006). Significance of a fragmented QRS complex versus a Q wave in patients with coronary artery disease. Circulation, 113, 2495–2501. 10.1161/CIRCULATIONAHA.105.595892 16717150

[anec12937-bib-0003] Das, M. K. , Maskoun, W. , Shen, C. , Michael, M. A. , Suradi, H. , Desai, M. , Subbarao, R. , & Bhakta, D. (2010). Fragmented QRS on twelve‐lead electrocardiogram predicts arrhythmic events in patients with ischemic and nonischemic cardiomyopathy. Heart Rhythm: the Official Journal of the Heart Rhythm Society, 7, 74–80. 10.1016/j.hrthm.2009.09.065 20129288

[anec12937-bib-0004] Fumimoto, T. , Ueyama, T. , Shimizu, A. , Yoshiga, Y. , Ono, M. , Kato, T. , Ishiguchi, H. , Okamura, T. , Yamada, J. , & Yano, M. (2017). Inferior J waves in patients with vasospastic angina might be a risk factor for ventricular fibrillation. Journal of Cardiology, 70, 271–277. 10.1016/j.jjcc.2016.12.003 28087290

[anec12937-bib-0005] Haraoka, K. , Morita, H. , Saito, Y. , Toh, N. , Miyoshi, T. , Nishii, N. , Nagase, S. , Nakamura, K. , Kohno, K. , Kusano, K. F. , Kawaguchi, K. , Ohe, T. , & Ito, H. (2010). Fragmented QRS is associated with torsades de pointes in patients with acquired long QT syndrome. Heart Rhythm: The Official Journal of the Heart Rhythm Society, 7, 1808–1814. 10.1016/j.hrthm.2010.09.008 20837161

[anec12937-bib-0006] JCS Joint Working Group . (2010). Guidelines for diagnosis and treatment of patients with vasospastic angina (coronary spastic angina) (JCS2008): Digest version. Circulation Journal, 74, 1745–1762. 10.1253/circj.CJ-10-74-0802 20671373

[anec12937-bib-0007] JCS Joint Working Group . (2014). Guidelines for diagnosis and treatment of patients with vasospastic angina (coronary spastic angina) (JCS 2013). Circulation Journal. 78, 2779–2801. 25273915 10.1253/circj.cj-66-0098

[anec12937-bib-0008] Kawana, A. , Takahashi, J. , Takagi, Y. , Yasuda, S. , Sakata, Y. , Tsunoda, R. , Ogata, Y. , Seki, A. , Sumiyoshi, T. , Matsui, M. , Goto, T. , Tanabe, Y. , Sueda, S. , Kubo, N. , Momomura, S.‐I. , Ogawa, H. , Shimokawa, H. , & on Behalf of the Japanese Coronary Spasm Association . (2013). Gender differences in the clinical characteristics and outcomes of patients with vasospastic angina–a report from the Japanese Coronary Spasm Association. Circulation Journal, 77, 1267–1274. 10.1253/circj.CJ-12-1486 23363662

[anec12937-bib-0009] Kawano, H. , & Ogawa, H. (2004). Endothelial dysfunction and coronary artery spasm. Current Drug Targets: Cardiovascular & Haematological Disorders, 4, 23–33. 15032650 10.2174/1568006043481301

[anec12937-bib-0010] Luo, G. , Li, Q. , Duan, J. , Peng, Y. , & Zhang, Z. (2020). The predictive value of fragmented QRS for cardiovascular events in acute myocardial infarction: A systematic review and meta‐analysis. Frontiers in Physiology, 11, 1027. 10.3389/fphys.2020.01027 33117185 PMC7574772

[anec12937-bib-0011] Marume, K. , Noguchi, T. , Kamakura, T. , Tateishi, E. , Morita, Y. , Miura, H. , Nakaoku, Y. , Nishimura, K. , Yamada, N. , Tsujita, K. , Izumi, C. , Kusano, K. , Ogawa, H. , & Yasuda, S. (2021). Prognostic impact of multiple fragmented QRS on cardiac events in idiopathic dilated cardiomyopathy. Europace, 23, 287–297. 10.1093/europace/euaa193 33212485

[anec12937-bib-0012] Matsue, Y. , Suzuki, M. , Nishizaki, M. , Hojo, R. , Hashimoto, Y. , & Sakurada, H. (2012). Clinical implications of an implantable cardioverter‐defibrillator in patients with vasospastic angina and lethal ventricular arrhythmia. Journal of the American College of Cardiology, 60, 908–913. 10.1016/j.jacc.2012.03.070 22840527

[anec12937-bib-0013] Morita, H. , Kusano, K. F. , Miura, D. , Nagase, S. , Nakamura, K. , Morita, S. T. , Ohe, T. , Zipes, D. P. , & Wu, J. (2008). Fragmented QRS as a marker of conduction abnormality and a predictor of prognosis of Brugada syndrome. Circulation, 118, 1697–1704. 10.1161/CIRCULATIONAHA.108.770917 18838563

[anec12937-bib-0014] Morita, H. , Watanabe, A. , Morimoto, Y. , Kawada, S. , Tachibana, M. , Nakagawa, K. , Nishii, N. , & Ito, H. (2017). Distribution and prognostic significance of fragmented QRS in patients with brugada syndrome. Circulation: Arrhythmia and Electrophysiology, 10, e004765. 10.1161/CIRCEP.116.004765 28314845

[anec12937-bib-0015] Myerburg, R. J. , Kessler, K. M. , Mallon, S. M. , Cox, M. M. , deMarchena, E. , Interian, A. , & Castellanos, A. (1992). Life‐threatening ventricular arrhythmias in patients with silent myocardial ischemia due to coronary‐artery spasm. New England Journal of Medicine, 326, 1451–1455. 10.1056/NEJM199205283262202 1574091

[anec12937-bib-0016] Nishizaki, M. (2017). Life‐threatening arrhythmias leading to syncope in patients with vasospastic angina. Journal of Arrhythmia, 33, 553–561. 10.1016/j.joa.2017.04.006 29255500 PMC5728714

[anec12937-bib-0017] Peters, S. , Trummel, M. , & Koehler, B. (2008). QRS fragmentation in standard ECG as a diagnostic marker of arrhythmogenic right ventricular dysplasia‐cardiomyopathy. Heart Rhythm: the Official Journal of the Heart Rhythm Society, 5, 1417–1421. 10.1016/j.hrthm.2008.07.012 18783995

[anec12937-bib-0018] Pietrasik, G. , & Zareba, W. (2012). QRS fragmentation: Diagnostic and prognostic significance. Cardiology Journal, 19, 114–121. 10.5603/CJ.2012.0022 22461043

[anec12937-bib-0019] Prinzmetal, M. , Kennamer, R. , Merliss, R. , Wada, T. , & Bor, N. (1959). Angina pectoris. I. A variant form of angina pectoris; preliminary report. American Journal of Medicine, 27, 375–388. 10.1016/0002-9343(59)90003-8 14434946

[anec12937-bib-0020] Schuller, J. L. , Olson, M. D. , Zipse, M. M. , Schneider, P. M. , Aleong, R. G. , Wienberger, H. D. , Varosy, P. D. , & Sauer, W. H. (2011). Electrocardiographic characteristics in patients with pulmonary sarcoidosis indicating cardiac involvement. Journal of Cardiovascular Electrophysiology, 22, 1243–1248. 10.1111/j.1540-8167.2011.02099.x 21615816

[anec12937-bib-0021] Yamashina, Y. , Yagi, T. , Namekawa, A. , Ishida, A. , Mibiki, Y. , Sato, H. , Nakagawa, T. , Sakuramoto, M. , Sato, E. , & Komatsu, J. (2014). Favorable outcomes of patients with vasospastic angina associated with cardiac arrest. Journal of Cardiology, 63, 41–45. 10.1016/j.jjcc.2013.06.011 23906527

[anec12937-bib-0022] Yonezu, K. , Shinohara, T. , Sato, H. , Hirota, K. , Kondo, H. , Fukui, A. , Teshima, Y. , Yufu, K. , Nakagawa, M. , & Takahashi, N. (2021). Role of fragmented QRS and shanghai score system in recurrence of ventricular fibrillation in patients with early repolarization syndrome. Annals of Noninvasive Electrocardiology, 26(6), e12873. 10.1111/anec.12873 34232529 PMC8588366

